# Dexamethasone Intravitreal Implant Is Active at the Molecular Level Eight Weeks after Implantation in Experimental Central Retinal Vein Occlusion

**DOI:** 10.3390/molecules27175687

**Published:** 2022-09-03

**Authors:** Lasse Jørgensen Cehofski, Anders Kruse, Mads Odgaard Mæng, Benn Falch Sejergaard, Anders Schlosser, Grith Lykke Sorensen, Jakob Grauslund, Bent Honoré, Henrik Vorum

**Affiliations:** 1 Department of Ophthalmology, Odense University Hospital, 5000 Odense, Denmark; 2 Department of Ophthalmology, Aalborg University Hospital, 9000 Aalborg, Denmark; 3 Department of Clinical Research, University of Southern Denmark, 5000 Odense, Denmark; 4 Department of Biomedical Research Laboratory, Aalborg University Hospital, 9000 Aalborg, Denmark; 5 Department of Cancer and Inflammation Research, University of Southern Denmark, 5000 Odense, Denmark; 6 Department of Clinical Medicine, Aalborg University, 9000 Aalborg, Denmark; 7 Department of Biomedicine, Aarhus University, 8000 Aarhus, Denmark

**Keywords:** retina, retinal vein occlusion, mass spectrometry, proteomics, proteome, dexamethasone, inflammation

## Abstract

Central retinal vein occlusion (CRVO) is a visually disabling condition resulting from a thrombus in the major outflow vessel of the eye. The inflammatory response in CRVO is effectively treated with a dexamethasone (DEX) intravitreal implant. Uncovering the proteome changes following DEX implant intervention in CRVO may identify key proteins that mediate the beneficial effects of DEX. In six Göttingen minipigs, CRVO was induced in both eyes with an argon laser using a well-established experimental model. The right eyes were treated with a DEX intravitreal implant (Ozurdex, Allergan), while the left control eyes received a sham injection. Eight weeks after DEX intervention, retinal samples were collected and analyzed with tandem mass tag-based mass spectrometry. DEX implant intervention resulted in the upregulation of peptidyl-prolyl cis–trans isomerase FKBP5 (FKBP5) and ubiquilin-4. Immunohistochemistry showed expression of FKBP5 in the nuclei in all cellular layers of the retina. Cell adhesion molecule 3, tumor necrosis factor receptor superfamily member 16, and trans-1,2-dihydrobenzene-1,2-diol dehydrogenase were downregulated following DEX intervention. The upregulation of the corticosteroid-sensitive protein FKBP5 suggests that the implant remained active at the molecular level after eight weeks of treatment. Future studies may investigate if FKBP5 regulates the efficacy and duration of the DEX implant.

## 1. Introduction

Central retinal vein occlusion (CRVO) is a visually disabling condition caused by a thrombus of the major outflow vessel of the eye, the central retinal vein [1,2]. If untreated, visual acuity following CRVO generally remains below 20/40. Macular edema is a frequent complication and the most common cause of visual loss in CRVO [3,4,5,6]. A number of processes drive the formation of macular edema. The occlusion of the central retinal vein results in increased resistance to blood flow in retinal arterioles with the closure of retinal capillaries and small arterioles, resulting in retinal hypoxia. Retinal hypoxia drives the increased production of vascular endothelial growth factor A (VEGF-A) and a complex inflammatory response mediated by interleukin (IL)-6, IL-8, IL-18, S100A12, fibronectin, galectin-3, and monocyte chemotactic protein-1 [3,4,5,6]. VEGF-A and the inflammatory response promote increased vascular permeability, resulting in the accumulation of fluid in the macular area of the retina [4,6].

Although macular edema following CRVO is effectively treated with a dexamethasone (DEX) intravitreal implant inhibiting VEGF-A signaling as well as the inflammatory response in CRVO [7,8,9], the retinal proteome in CRVO following DEX implant intervention remains largely unstudied [10]. Knowledge of the proteins that mediate the beneficial effects of the DEX implant may be useful in developing novel therapeutic strategies and in improving existing treatments. Furthermore, there is very limited knowledge about retinal proteome changes after long-term treatment with DEX implant intervention in CRVO [10].

Retreatment is often required in the management of macular edema secondary to CRVO [7,9], but the timing of retreatment remains a challenge in any retina clinic. Studying the retinal proteome after eight weeks of treatment may provide important insights into the efficacy and duration of the DEX implant [8]. Knowledge about proteome changes at eight weeks after DEX implant intervention may be valuable in decision-making related to retreatment. A period of eight weeks of treatment is fairly difficult to achieve in models based on large animals, but our recent advances in an experimental porcine CRVO model [5] now allow for treatments up to eight weeks.

Retinal tissue exposed to CRVO is generally only available in animal models. Porcine eyes are similar to human eyes in terms of vascularization and size, which renders them well-suited for studies of retinal vascular disease [10,11,12].

In the study presented, a DEX intravitreal implant was tested in a well-established porcine model of laser-induced CRVO [5], which is useful in proteomic studies due to its non-invasive nature. Advanced proteomic techniques were used to study large-scale retinal protein changes following DEX implant intervention in the CRVO model.

## 2. Results

### 2.1. Evaluation of Experimental CRVO Model

**Figure 1:**
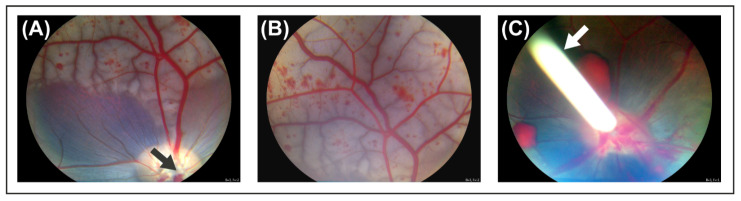
Funduscopic images obtained four days after induced CRVO. (**A**) Retinal hemorrhages and dilation of retinal veins are observed upstream of the site of occlusion. Black arrow: site of occlusion. (**B**) Fundus image of the peripheral retina. Multiple retinal hemorrhages are observed. (**C**) Dexamethasone (DEX) implant injected into eye with experimental CRVO. White arrow: DEX implant.

**Figure 2:**
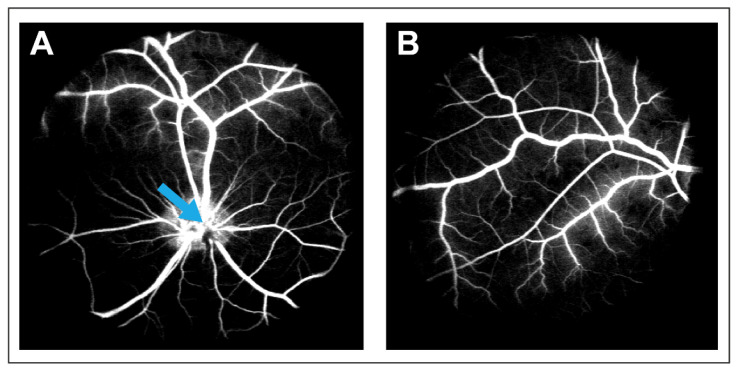
Fluorescein angiography performed four days after CRVO was induced. (**A**) Delayed venous filling and leakage can be observed in the CRVO model. Blue arrow: Site of occlusion. (**B**) Leakage in the peripheral retina observed on angiography following CRVO.

CRVO was successfully induced with argon laser in both eyes of Göttingen minipigs (Figure 1). Fluorescein angiography performed three days and six weeks after induced CRVO showed delayed filling of retinal branch veins and leakage around retinal veins (Figure 2). DEX implants were implanted in the right eye of each animal, while the left eye received a sham injection. The duration of the treatment was eight weeks.

### 2.2. Dexamethasone (DEX) Intervention in CRVO Model

**Figure 3:**
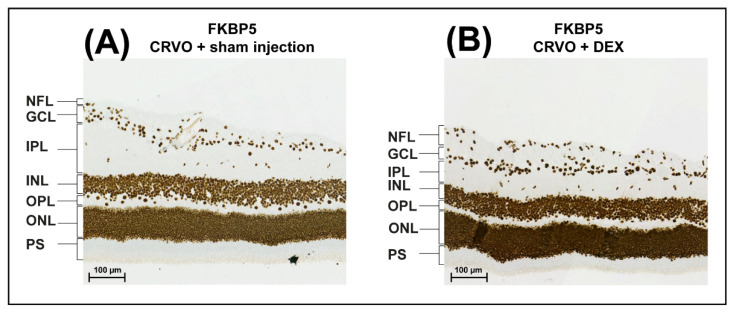
Immunohistochemistry. (**A**,**B**) Peptidyl-prolyl cis-trans isomerase (FKBP5) was expressed in all retinal cellular layers regardless of DEX treatment. The staining for FKBP5 was stronger following DEX treatment compared with sham injection. The upregulation of FKBP5 was strongest in the nuclei of cells in the inner nuclear layer and the outer nuclear layer. The upregulation of FKBP5 was strongest in the central areas of the retina close to the optic nerve head. Scale bar = 100 µm. Reaction color: brown. Abbreviations: NFL: nerve fiber layer; GCL: ganglion cell layer; IPL: inner plexiform layer; INL: inner nuclear layer; OPL: outer plexiform layer; ONL: outer nuclear layer; PS: photoreceptor segments.

Tandem mass tag based mass spectrometry was used to compare CRVO + DEX (*n* = 5) with CRVO + sham (*n* = 5). The data output from MaxQuant is available in the Supplementary Tables (Tables S1 and S2). A total of 2749 proteins were successfully identified and quantified in the retinal samples (Supplementary Material). A total of five proteins were significantly regulated following DEX implant intervention in CRVO (Table 1). Upregulated proteins included peptidyl-prolyl cis-trans isomerase (FKBP5) (fold change = 1.47; *p* = 0.047) and ubiquilin-4 (fold change 1.35; *p* = 0.014) (Table 1). Immunohistochemistry showed that FKBP5 was expressed in the nuclei in all retinal cell layers (Figure 3). Modest upregulation of FKBP5 was observed in the nuclei of retinal cells following DEX intervention, especially in the inner nuclear layer and the outer nuclear layer (Figure 3). The upregulation of FKBP5 was generally higher in the central parts of the retina close to the optic nerve head compared with the peripheral retina.

Significantly downregulated proteins included cell adhesion molecule 3 (CADM3) (fold change = 0.69; *p* = 0.049), tumor necrosis factor receptor superfamily member 16 (fold change = 0.68; *p* = 0.021), and trans-1,2-dihydrobenzene-1,2-diol dehydrogenase (fold change = 0.61 *p* = 0.036) (Table 1).

## 3. Discussion

### Proteome Changes in Experimental CRVO Following DEX Implant Intervention

Proteomic analysis of the retina is associated with a number of limitations [11]. We previously reported that proteome changes often occur in specific retinal layers [5,8]. Due to the multi-layered structure of the retina, observed proteome changes in the retina may be moderate when the entire neuroretina is collected for analysis. The isolation of particular retinal cells or layers may potentially cause higher fold changes [11]. As the presented study is the first of its kind, we were not able to predict the retinal layers in which the proteome changes would occur. Another aspect to consider is the constant, slow release of DEX from the implant. A total of five proteins were regulated following DEX intervention in CRVO, with fold changes ranging between 0.61–1.47. The modest changes may be ascribed to the sustained delivery with a slow, but constant, release of DEX from the implant as well as the proteome analysis being conducted on the entire neuroretina.

While protein changes were modest, it is interesting that findings from a previous proteome study of the DEX implant were reproduced. We previously identified an upregulation of FKBP5 and a downregulation of CADM3 in a proteome study where the DEX implant intervention was studied in a model of experimental branch retinal vein occlusion (BRVO) [8], a subtype in which a sector of the retina is affected by retinal vein occlusion [13]. In our previous study of BRVO, the duration of DEX implant intervention was two weeks [8]. Our results strongly suggest that FKBP5 and CADM3 are corticosteroid-sensitive proteins in retinal vein occlusion, as the regulation of the proteins has been identified in two individual proteome studies of the DEX implant.

As expected, FKBP5 was localized in the nuclei of the retinal nuclear layers. The staining for FKBP5 was particularly strong close to the optic nerve head following treatment with the dexamethasone implant. Thus, staining for FKBP5 was particularly strong in the area close to the occlusion. While the immunohistochemistry shows the retinal area close to the occlusion, quantification with mass spectrometry was performed on the entire retina. This may explain why some discrepancy between immunohistochemistry and mass spectrometry was observed. FKBP5 belongs to the immunophilin family of proteins [14,15]. It serves as a chaperone for the corticosteroid receptors GBα and GBβ, and is involved in the translocation of these receptors into the nucleus [16]. The upregulation of FKBP5 is consistent with an activated corticosteroid response [17], indicating that the DEX implant remains active eight weeks after the injection of the implant. As the results indicate that the DEX implant is still active at the molecular level after eight weeks, our results may support retreatment later than two months. Increased FKBP5 expression has also been reported in the adult retinal pigment epithelium cell line (ARPE)-19 and in choroidal vascular epithelial cells treated with DEX [14,18]. While the upregulation of FKBP5 reflects an activated corticosteroid response, FKBP5 is also a suppressor of the corticosteroid receptor [19,20]. Therefore, future studies may investigate if FKBP5 regulates the efficacy and duration of the DEX intravitreal implant.

CADM3 belongs to the immunoglobulin superfamily of type I transmembrane proteins with three extracellular immunoglobulin-like loop domains and a cytoplasmic tail with two protein–protein interaction domains [21]. CADM3 mediates Ca^2+^-independent cell adhesion [22]. Retinal CADM3 is expressed in photoreceptors, bipolar cells, retinal ganglion cells, and the optic nerve [23]. Anti-inflammatory treatment has previously been reported to result in the downregulation of CADM3. In cerebrospinal fluid from patients with polyarthritis, a downregulation of CADM3 has been reported following TNF-α blockage [24]. A similar anti-inflammatory effect following DEX intervention, which downregulated CADM3, was observed in our study.

## 4. Materials and Methods

### 4.1. Animal Preparation

The study was approved by the Danish Animal Experiments Inspectorate, permission no. 2019-15-0201-01651. Göttingen minipigs were housed under a 12 h light/dark cycle, and general anesthesia and topical anesthesia were performed as previously described [25]. The dilation of the pupils was performed with tropicamide 1.0% (Bausch & Lomb, Bridgewater, NJ, USA) and phenylephrine 10% (Bausch & Lomb, Bridgewater, NJ, USA).

### 4.2. Experimental CRVO

Experimental CRVO was induced in both eyes of six Göttigen minipigs using a well-established model of experimental CRVO as described in detail in a previous paper [5]. Briefly, CRVO was induced in proximity to the optic nerve head with a standard argon laser (532 nm) given by indirect ophthalmoscopy using a 20D lens. The laser settings were 400 mW per application and an exposure time of 550 ms. A total of 30–40 laser applications were used per occlusion. By applying the laser directly onto retinal veins close to the optic nerve head, thrombotic material was directed toward the optic nerve head and the lamina cribrosa. Experimental CRVO was considered to be successfully induced when the stagnation of venous blood and development of flame-shaped hemorrhages appeared.

A DEX intravitreal implant of 0.7 mg (Ozurdex, Allergan, Søborg, Denmark) was injected into the right eyes of the animals, while the left eyes, which served as controls, were given an intravitreal injection without the injection of an implant (sham). CRVO was verified with fluorescein angiography three days after CRVO was induced and repeated after six weeks.

Eyes were enucleated eight weeks after induced CRVO and dissected on ice under a microscope. The anterior segment was removed by cutting 4 mm posterior to the limbus followed by the removal of the vitreous body, which was aspired into a 5 mL syringe. In the eyes used for proteomic analysis, CRVO + DEX (*n* = 5) and CRVO + sham (*n* = 5), the neurosensory retina was peeled from the RPE/choroid complex with tweezers and stored at −80 °C until further use. In the eyes intended for immunohistochemistry, CRVO + DEX (*n* = 1) and CRVO + sham (*n* = 1), complexes consisting of the neurosensory retina, RPE/choroid complex, and sclera were excised. The animals were euthanized as soon as enucleation had been completed.

### 4.3. Sample Preparation for Mass Spectrometry

Eyes from five animals were used to study proteome changes following DEX implant intervention in CRVO, comparing CRVO + DEX (*n* = 5) vs. CRVO + sham (*n* = 5) with proteomic analysis by tandem mass tag (TMT) based mass spectrometry. Sample preparation was performed with the suspension-trapping method [26] using S-Trap mini columns from Protifi (Farmingdale, NY, USA), as described in detail in a previous study [6]. TMT-based mass spectrometry was performed essentially as described in a recent paper [27]. Eleven micrograms from each sample were used for TMT labeling with TMT10-plex kit (Lot number: RG234624A, Thermo Scientific, Waltham, MA, USA). High-pH reversedphase peptide fractionation into 8 fractions was performed as described in a previous study [28].

### 4.4. Quantification with Tandem Mass Tag-Based Mass Spectrometry

One microgram of each fraction was loaded for each run onto a Dionex UltiMateTM 3000 RSLC nanosystem coupled to an Orbitrap Fusion mass spectrometer (Thermo Scientific, Waltham, MA, USA) equipped with an EasySprayTM ion source. Liquid chromatography and mass spectrometry with the TMT synchronous precursor selection MS^3^ mode were performed essentially as described in a recent paper [27], with an AGC target of 2 × 105 and a maximal injection time of 70 ms. Three replicas of each fraction were analyzed.

The raw files were used for database search using MaxQuant software version 1.6.6.0 (Max Planck Institute of Biochemistry, Martinsried, Germany; https://maxquant.net/maxquant/) [29]. Raw data files were searched against the Uniprot *Homo sapiens* database [30] and the UniProt *Sus scrofa* with settings reported in a previous paper [8,27]. Unfiltered results from the database search are available in Supplementary File S1.

### 4.5. Filtration of Proteins and Statistics

The ProteinGroups file was uploaded to Perseus version 1.6.2.3 (Max Planck Institute of Biochemistry, Martinsried, Germany; https://maxquant.net/perseus/) [31]. The quantitative values were log_2_-transformed. The successful identification of a protein required at least two unique peptides. Proteins were required to be successfully identified and quantified in 100% of the samples. Technical replicates were averaged. Student’s *t*-test was performed in Perseus to compare CRVO + DEX (*n* = 5) vs. CRVO + sham (*n* = 5). Proteins were considered significantly regulated when *p* < 0.05.

### 4.6. Immunohistochemistry

Eyes from one animal were used to compare CRVO + DEX (*n* = 1) vs. CRVO + sham (*n* = 1). Complexes consisting of the retina, choroid, and sclera were fixed in formalin for 24 h. The formalin solution was removed. The tissue was stored in a PBS solution at 4 °C until further use. Immunohistochemistry was performed as previously described [27] with a polyclonal anti-FKBP5 middle-region antibody (MBS3211817, MyBiosource, San Diego, CA, USA). Image acquisition was performed as described in a recent article [27].

## 5. Conclusions

The proteome analysis showed modest changes following DEX intervention, reflecting the slow release of DEX from the implant. The upregulation of the corticosteroid-sensitive protein FKBP5 eight weeks after DEX intervention suggests that the DEX implant was active eight weeks after intervention in CRVO. As FKBP5 is also a suppressor of the corticosteroid receptor, future studies may investigate if FKBP5 regulates the efficacy and duration of the DEX intravitreal implant. DEX intervention resulted in the downregulation of CADM3 which may contribute to an anti-inflammatory response.

## Figures and Tables

**Table 1:** Regulated proteins following dexamethasone (DEX) intervention in experimental CRVO.

Protein ID	Protein Name	Gene Name	*p*-Value	Ratio DEX/Sham
Q13451	Peptidyl-prolyl cis-trans isomerase FKBP5 (FKBP5)	FKBP5	0.047	1.47
Q9NRR5	Ubiquilin-4	UBQLN4	0.014	1.35
Q8N126-3	Cell adhesion molecule 3 (CADM3)	CADM3	0.049	0.69
P08138-2	Tumor necrosis factor receptor superfamily member 16	NGFR	0.021	0.68
Q9TV69	Trans-1,2-dihydrobenzene-1,2-diol dehydrogenease	DHDH	0.036	0.61

## Data Availability

Please see the Supplementary Tables (Tables S1 and S2).

## Supplementary Materials

The following supporting information can be downloaded at: https://www.mdpi.com/article/10.3390/molecules27175687/s1, Table S1: Unfiltered results from database search; Table S2: All successfully identified proteins.
